# Quantitative Infrared Thermography to Evaluate the Humidification of Lightweight Concrete

**DOI:** 10.3390/s20061664

**Published:** 2020-03-17

**Authors:** Eva Barreira, Ricardo M.S.F. Almeida, Maria L. Simões, Daniela Rebelo

**Affiliations:** 1 CONSTRUCT-LFC, Department of Civil Engineering, Faculty of Engineering, University of Porto, Rua Dr. Roberto Frias s/n, 4200-465 Porto, Portugal; ralmeida@estv.ipv.pt (R.M.S.F.A.); lurdes.simoes@fe.up.pt (M.L.S.); up201306112@fe.up.pt (D.R.); 2 Department of Civil Engineering, Polytechnic Institute of Viseu, Campus Politécnico de Repeses, 3504-510 Viseu, Portugal

**Keywords:** infrared thermography, quantitative analysis, moisture, descriptive statistics, thermal images subtraction, thermal index

## Abstract

Moisture is one of the major causes of problems in buildings, and it can compromise their performance. Infrared thermography (IRT) is a non-destructive testing technology that can be used to assess the humidification phenomenon and, thus, prevent some of the problems caused by moisture. The images obtained by IRT reflect the thermal patterns of the surface under study and can be evaluated using a quantitative approach, which allows not only the traditional visualization of the thermal patterns but also quantification of surface temperatures and/or their differences. The relevance of this work is related to the discussion of the strengths and weaknesses of several methods to quantitatively assess the humidification phenomenon using IRT. For that purpose, the partial humidification by the bottom surface of a lightweight concrete specimen was considered as a case study. To evaluate the thermal gradients, the evolution of the thermal imaging throughout the measurement period and the definition of the areas particularly affected by moisture, a methodology that included a pre-processing phase for data reduction, followed by a data processing phase, were implemented. In the data processing, different statistical and numerical methods were tested. The results of the statistical descriptive analysis highlighted the time variation of the surface temperature, both when considering the entire specimen and when considering only specific areas. The variability of the temperatures at certain moments of the experiment could be observed in the box-plot representation. The image subtraction proved to be an interesting technique to quantify the temperature differences if the first image was used as reference. A thermal index, TI, was proposed to assess the cooling rate. The index highlighted the initial instant when the effect of moisture on the surface temperature was detectable.

## 1. Introduction

Moisture is one of the main causes of pathologies in buildings. If not detected and controlled at the early stage, it can compromise the performance of buildings in terms of durability, mechanical behavior, and appearance. Last but certainly not least, it can have a considerable impact on user health.

Infrared thermography (IRT) can be a very useful diagnostic tool to help understand the humidification phenomenon. It measures surface temperature using a detector that captures infrared radiation and converts it into electrical signals. Thermal images or thermograms are the final result of the measurement. These images reflect the thermal patterns through a scale of colors or tones, and they can be evaluated using a qualitative and/or quantitative approach [1,2].

This non-contact and non-destructive technique was used to assess buildings for a couple of decades [1,2,3,4,5,6]. Particularly, when used to evaluate moisture problems [7,8,9,10,11,12], thermal patterns can result from (a) evaporative cooling at the moist area as evaporation decreases surface temperature [13,14,15,16,17,18,19], (b) reduced thermal resistance of the wet materials [20,21], and (c) increased heat storage capacity of the moist material [22,23].

Although some previous studies using quantitative analysis of the thermal images can be found, this topic is still evolving. Several authors reported that, in this kind of analysis, special attention should be paid not only when the thermal images are being collected but also during the data treatment [16,24]. 

Descriptive statistics can be useful for image interpretation, providing additional information, which is often impossible to detect in the qualitative analysis. Several statistical indicators can be used. Normally, in IRT, the third moment or skewness, which represents the lack of symmetry of the dataset, and the fourth moment or kurtosis, which characterizes the dataset distribution compared to a normal distribution, are the indicators of interest. These indicators are used to summarize the original sequence of temporal temperature profiles into a single image that includes all the relevant information [25]. For example, Sfarra et al. [26] used high-order statistics to assess the state of conservation of the historical mural painting Madonna con Bambino. A set of cracks was detected using a combined three-dimensional (3D) skewness image and the kurtosis results.

Several authors also used indices to quantitatively analyze the results of the thermographic measurements. Tavukçuoğlu and Grinzato [27] and Grinzato et al. [16] proposed a new parameter, the evaporative thermal index (ETI), defined as the difference between the dry and moist surface temperature divided by the dry one. With this parameter, the authors could estimate the evaporation rate on the surface and calculate the critical moisture content of materials without considering the distribution of moisture inside the sample. The same authors [16] presented another index that was used when the saturation at the surface was not reached, decreasing the evaporation rate. This parameter was suggested to assess moisture in building components and not only in building materials.

The saturation thermal index (STI) was defined as the ratio of the surface temperature difference between the moist and dry wall to the temperature difference of the same porous material in saturation and dry conditions. With this index, the authors could define an accurate map of the walls because using a normalized temperature difference allowed them to restrict the uncertainty related to emissivity and to the influence of the environment [28]. In the research carried out by Madruga et al. [29], the relationship between wood and its degree of humidity was analyzed using IRT to retrieve thermal parameters of the wood related to the amount of water added to the samples.

Barreira et al. [6] introduced the pressurization thermal index (PTI) to assess the effect of leakage points in buildings. This index traduces the ratio of the difference between the final and initial superficial temperature and the final pressure difference. According to the authors, it quantifies the effect of the air leakage on the superficial temperature and it is proportional to the temperature gradient between the exterior air temperature and the initial temperature of the surface. 

Image subtraction is another technique commonly used in the quantitative analysis of thermograms. This method of image processing allows identifying small changes in the surface temperature, reducing or even eliminating the influence of external factors as environmental reflections. If applied consecutively, the method allows visualizing the variation in time of a thermal anomaly caused by a transient phenomenon [24]. In this method, a thermal image taken at a specified moment is used as a reference to highlight details in other images by finding pixel-by-pixel temperature differences between them [23,30,31]. The image subtraction can be performed in two ways: by subtracting the image chosen as reference from the sequence of thermal images or by subtracting each image from the previous one [24].

Lerma et al. [23] used image subtraction to create a cooling map of a set of walls, using the images corresponding to the maximum and minimum temperatures. Despite the advantages of the technique, the authors stated that the variations of the ambient temperature during the measurement period may bias the results. Edis et al. [30] reported difficulties in finding a good correlation between the results of images subtraction and those obtained with a moisture detector. For the subtraction, the first and the last thermal images taken during a time-dependent IRT inspection were used as references for the morning and late-afternoon calculations, respectively. Tavukçuoğlu et al. [32] successfully used image subtraction to highlight structural cracks in historic masonry structures.

This work presents a discussion regarding the strengths and weaknesses of several different statistical and numerical tools to quantitatively assess the humidification phenomenon using IRT. For that purpose, the partial humidification by the bottom surface of a lightweight concrete specimen was considered as the case study. Although not moisture related, active IRT was previously successfully applied to detect defects in concrete [33].

## 2. Methodology

### 2.1. Test Procedures

**Figure 1:**
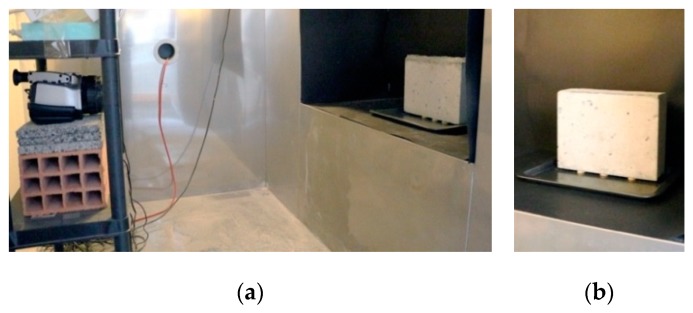
(**a**) Test set-up; (**b**) partial humidification of the specimen by the bottom.

A lightweight concrete specimen was used as a case study. Table 1 shows the main characteristics of the material under study [34]. The test was performed inside a climatic chamber under stable conditions (20 ± 0.5 °C and 60% ± 2%). The specimen was partially humidified by the bottom surface, after being previously dried in an oven at 110 ± 5 °C until mass stabilization was reached (mass variation of 0.1% of the total mass of the specimen, when measured over 24 h). The humidification occurred by placing the specimen for 24 hours inside a tank filled with tap water to a level of 5 ± 2 mm above the base of the specimen. This level was maintained during the entire humidification period (Figure 1).

The thermal images were taken every 5 min during the first 8 h and every 10 min during the remaining 16 h. A passive approach was used as no artificial thermal excitation was applied. The infrared (IR) camera remained in the same position during the entire test, and the climatic chamber was lined with black cardboard to avoid reflections. No operator was inside the climatic chamber as the camera was programmed to capture the images automatically. All compensations imposed by the camera were carried out before the beginning of the test. The specifications of the IR camera are displayed in Table 2.

### 2.2. Imaging Processing

**Figure 2:**
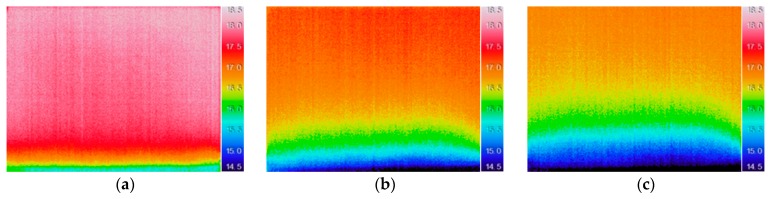
Thermal images of the entire specimen: (**a**) t = 0 h (immediately after the beginning of humidification); (**b**) t = 4 h; (**c**) t = 24 h.

After the 24 h of the humidification period, 193 thermal images were available for data processing. Three thermal images of the entire specimen (t = 0 h, t = 4 h, and t = 24 h) are presented in Figure 2. The humidification phenomenon is clearly visible in the thermal images. The lower temperatures (blue, green, and yellow colors) correspond to the moist areas near the bottom and are due to evaporative cooling. Over time, it is possible to see the moist area increasing as a result of rising dampness by capillarity through the porous material.

**Figure 3:**
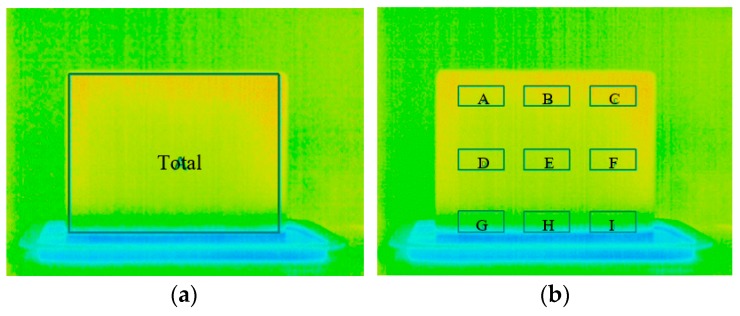
Definition of the boxes used: (**a**) box limiting the entire specimen with 189 × 143 pixels (for step I); (**b**) nine identical boxes with 42 × 19 pixels defined on the images (for step II).

Three different techniques were used to qualitatively evaluate the results: (A) descriptive statistics (statistical measures, histograms, and box plots); (B) image subtraction, which was used to highlight the surface temperature variations between measurement periods; (C) thermal index, traducing the variation rate of the surface temperature. To implement these techniques, two different methodologies were applied: (i) one box limiting the entire specimen (189 × 143 pixels) was selected on the thermal image (Figure 3a); (ii) nine identical boxes were defined on the images, three at the top of the specimen (boxes A, B, C in Figure 3b), three in the middle (boxes D, E, F in Figure 3b), and three at the bottom (boxes G, H, I in Figure 3b). All nine boxes were defined with 42 × 19 pixels.

**Figure 4:**
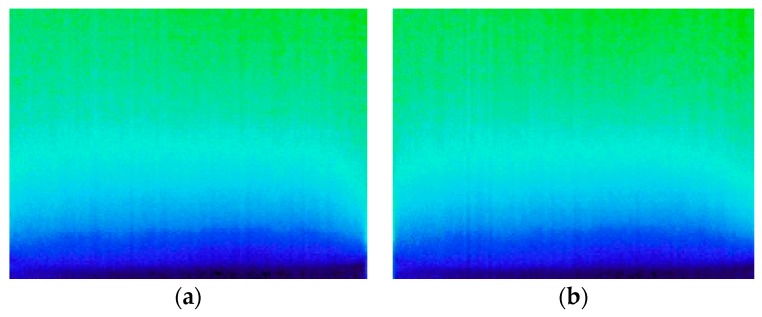
Thermal image of specimen during the humidification process: (**a**) t = 6 h; (**b**) t = 7 h.

The application of these methodologies required a pre-processing phase (Phase 1) in which a sample reduction was carried out. The initial set of 193 images was considered too high for the purpose of this research. For that reason, after a qualitative evaluation of the images pointing to identical information in several of them (Figure 4), a procedure to reduce the dataset was carried out. This procedure included the following steps:

Step I The average surface temperature of the entire specimen was considered, and the statistic *t*-test was used to compare consecutive thermal images. With Step I, a reduction by one-half was expected.
Step II Step II Only the average surface temperature of the boxes G, H, and I was considered, as the lower area of the specimen was deemed to be the most relevant in the phenomenon. Once more, the statistic *t*-test was used to compare consecutive thermal images aiming for a reduction by one-quarter.

Phase 2 started with the analysis based on the descriptive statistics, considering both the box limiting the total area of the specimen (Figure 3a) and the nine boxes (Figure 3b). The following descriptive statistical measures were calculated: mean, median, standard deviation, maximum, minimum, and skewness coefficient. Based on these values, the distribution of the surface temperatures over the humidification process was analyzed. In addition to the statistical descriptive analysis, some graphical distribution illustrations, such as histograms and box plots, were added to visually compare the temperature variability over time when considering the top, middle, and bottom of the specimen.

Image subtraction was carried out considering, as a reference, the first thermal image taken immediately after the humidification period began (Figure 2a) and by subtracting each image from the previous one. The temperature matrix of each thermal image was exported, and, after the pixel-by-pixel subtraction procedure, the new images were created using specific imaging software.

To assess the cooling rate of the specimen, a thermal index, *TI*, was defined according to Equation (1), varying between 0 and 1, in which 0 corresponds to the beginning of the humidification (no cooling effect) and 1 corresponds to the end of the experiment (maximum cooling effect). *TI* was used applied to the entire specimen and to the boxes.

(1) $$TI_{i}=\frac{\Delta T_{i}}{\Delta T_{max}}=\frac{T_{0}-T_{i}}{T_{0}-T_{f}},$$

where *T*_0_ is the average surface temperature at the beginning of the humidification, *T_i_* is the average surface temperature of thermal image *i*, and *T_f_* is the average surface temperature at the end of the experiment.

**Figure 5:**
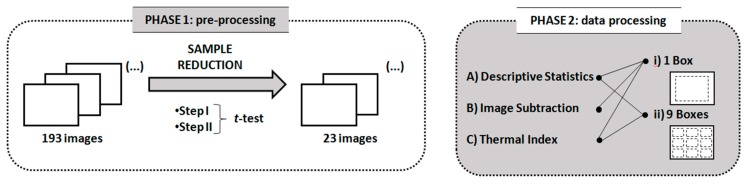
Methodology.

Figure 5 schematically illustrates the methodology of the research.

## 3. Results

### 3.1. Phase 1—Pre-Processing

#### 3.1.1. Sample Reduction

In the first step of the sample reduction, the statistic *t*-test was applied to compare sample means of the 193 thermal images. The average temperature of every two consecutive thermal images of the time sequence was compared to understand if they were statistically different. In the first attempt, a significance level of 5% was considered, but it proved to be a particularly demanding criterion as the results revealed that all images were statistically different. For that reason, a new criterion was implemented, establishing a *t*-value higher than 20 as the rejection limit. This procedure resulted in a 70% reduction, corresponding to 58 thermal images remaining.

The bottom layer of the specimen is the most relevant for the phenomenon; therefore, in the second step of the reduction procedure, only the temperature of boxes G, H, and I was analyzed. The average temperature of the three boxes of consecutive thermal images was compared, and the rejection criterion was a *t*-value higher than 10. This procedure resulted in a 60% reduction, and, after this step, 23 thermal images were selected to characterize the time-variability of the temperature.

**Figure 6:**
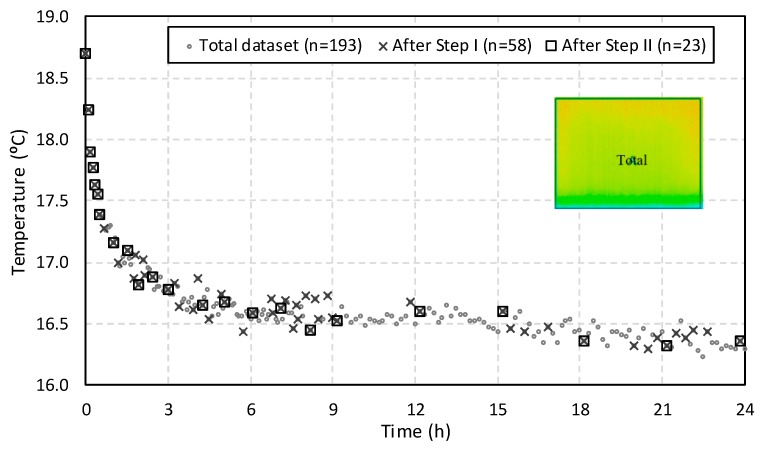
Sample reduction due to pre-processing phase.

Figure 6 illustrates the average temperature of the entire specimen (Figure 3a) before (total dataset) and after carrying out the two phases (after Step I and after Step II) of the reduction procedure. During the first hour, most of the thermal images were maintained after the pre-processing phase, because the changes on the surface temperature occurred rapidly, resulting in significant differences even between consecutive images. After Step I, a reduction of 31% was accomplished, increasing up to 38% after Step II. The temperature differences occurred due to a combined effect of water evaporation at the surface as a result of the humidification phenomenon and thermal adjustment to the environment. The analysis presented in the sections below proves the importance of the latter.

Between the first and ninth hours, after Step II, the number of thermal images was considerably reduced, because the absorption and evaporative phenomena stabilized, decreasing the number of relevant changes on the surface temperature between consecutive images. In this period, after Step I, 32 of the initial 90 images were maintained, decreasing to only nine after Step II. This stabilization effect was even more pronounced between the ninth hour and the end of the experiment (t = 24 h), as only six thermal images were maintained after Step II (93% reduction).

#### 3.1.2. Surface Temperature over Time

**Figure 7:**
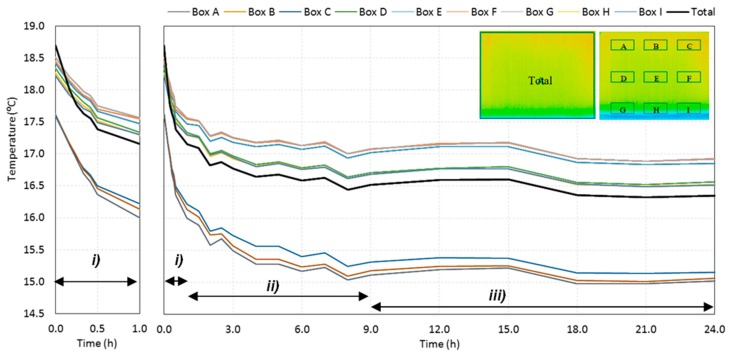
Evolution over time of the average temperatures of the specimen and of the nine boxes: (**i**) first hour, when the temperature drop was sharp; (**ii**) between 1 and 9 h, when the temperature kept decreasing, although at a much slower rate; (**iii**) after 9 h, when the temperature remained almost constant until the end of the test.

The time-variation of the average temperature of the entire specimen and of the nine boxes is presented in Figure 7. The graph includes data from the 23 thermal images selected after Step II of the pre-processing phase. The results showed that the average surface temperature of the specimen during the humidification period decreased, starting with its maximum value of about 19 °C and decreasing down to 16.3 °C at the end of the process. Three periods can be identified throughout the test: (i) in the first hour, the temperature drop was sharp; (ii) between 1 and 9 h, the temperature kept decreasing, although at a much slower rate; (iii) after 9 h, the temperature remained almost constant until the end of the test.

The results of the average temperature in the boxes confirmed a clear stratification of the temperature throughout the test as boxes at the same level had similar behavior. The lowest temperatures occurred at the bottom of the specimen and, after stabilization (period (iii)), the difference for the temperatures at the middle was around 1.5 °C. Comparing the temperatures at the middle and at the top, the difference was not that large (only around 0.5 °C). This was an expected result as the effect of evaporation was more important at the bottom, due to the higher amount of water available. Only a small amount of the absorbed water reached the middle of the specimen, thus decreasing the evaporation and reducing the temperature differences along the height.

While a homogeneous profile was observed in the rest of the specimen, if one analyzes the bottom layer, a small difference can be found when comparing box G with boxes H and I. In fact, the average temperature at the left corner was slightly higher than the rest of the specimen at this level. This may be related to asymmetric convective conditions inside the climatic chamber that enhanced evaporation in boxes H and I, decreasing their temperature.

### 3.2. Phase 2—Data Processing 

#### 3.2.1. Statistical Descriptive Analysis

The potential of descriptive statistical analysis was tested for the identification of temperature heterogeneities in the specimen, including the location of the areas where major and minor changes occurred and the temperature variability over time.

**Figure 8:**
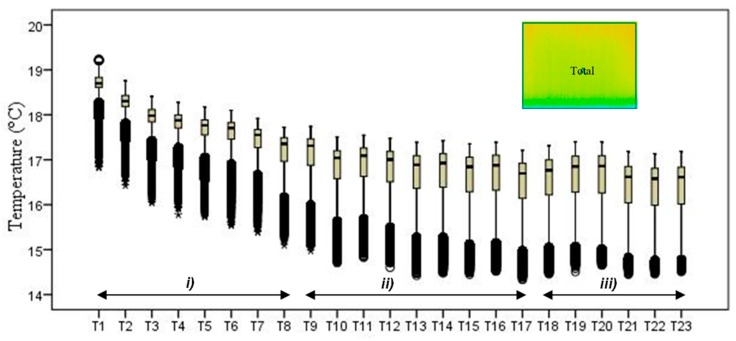
Box-plot representation of the surface temperatures of the entire specimen: (**i**) first hour, when the temperature drop was sharp; (**ii**) between 1 and 9 h, when the temperature kept decreasing, although at a much slower rate; (**iii**) after 9 h, when the temperature remained almost constant until the end of the test.

The first example is presented in Figure 8, where the time-variation of the surface temperature of the entire specimen can be observed using a box-plot representation. This method, in addition to exposing the temperature decrease throughout the experiment, allows analyzing the variability of the data. In fact, a substantial increase in the variability can be observed, as more compact boxes were obtained in the first hour (period (i)). This was due to the increase in the wet area, inducing higher differences between the temperatures on the moist area (colder temperatures) and those on the dry surface (higher temperatures). Another important aspect was the outliers found in the thermal images, all corresponding to lower temperatures. While, in the beginning of the experiment, the number of outliers was very high, they tended to decrease throughout the test. In the first thermal images, the outliers corresponded to the bottom layer of the specimen where the liquid water in the tank significantly affected the temperature. As humidification occurred and the water level raised, the effect of this layer was less obvious due to the increase in the evaporation region, thus reducing the number of outliers.

**Figure 9:**
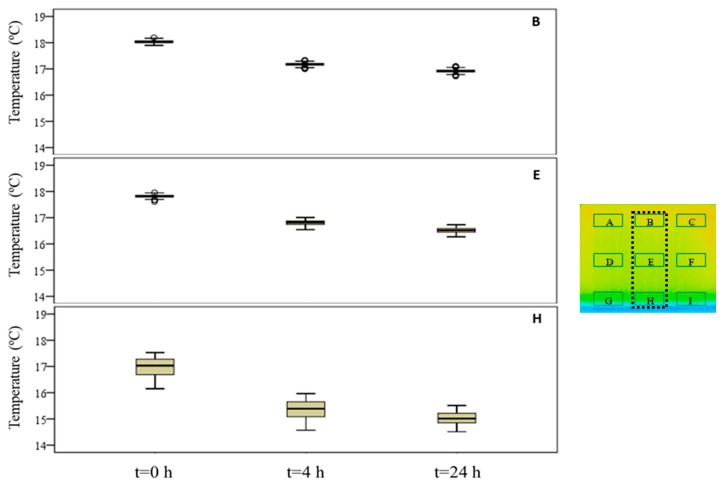
Box-plot representation of the surface temperatures of boxes B, E, and H.

The box-plot representation can also be applied to the individual boxes, for instance, to highlight the increase in temperature stratification throughout the experiment (Figure 9). Analyzing boxes B, E, and H, located in the central region of the specimen, it is possible to conclude that the temperature variations were higher in box H due to the effect of water evaporation. The temperature range in box H, at different periods in time, pointed to largest variations in the first hours, tending to stabilize at the end of the humidification. In boxes B and E, the temperature was more homogeneous, without large fluctuations. However, in box E, the temperature spread was slightly higher after 24 h, indicating that the rising water reached the middle layer of the specimen.

Descriptive statistical analysis could be applied to a detailed evaluation of a specific moment of the experiment. Table 3 presents an example using the temperature values of the thermal image taken at the end of the humidification period. Several statistical indicators were calculated separately for each box and for the entire image, namely, mean, median, standard deviation, skewness coefficient, maximum, and minimum. Additionally, two graphical representations were also included box plot and histogram.

The results obtained for the nine boxes highlight the temperature stratification throughout the specimen. The surface temperature in each of the three levels was generally homogeneous, as mean, median, standard deviation, maximum, and minimum were identical. The exception was the previously referred slight asymmetry found in the bottom layer, with a trend to lower temperatures in the right side of the specimen (box I). The value of the skewness coefficient, and the box-plot and the histogram representations showed an almost symmetrical distribution of the temperature within each box. However, it is possible to observe that the heterogeneity (right asymmetry) of the temperature was more pronounced in the middle layer because, at the end of the experiment, it corresponded to a transition zone affected by the water level.

If one analyzes the result for the entire specimen, the mean and median values were not very useful. On the other hand, the standard deviation indicated a relevant spread around the mean value of the temperature. The difference between the maximum and minimum values also pointed to a relevant temperature gradient throughout the height of the specimen at the end of the experiment (ΔT_max_ = 2.7 °C). The skewness coefficient and the graphical representations confirmed this asymmetry.

#### 3.2.2. Analysis Based on Image Subtraction

Data processing by image subtraction was performed considering the 23 thermal images and two different procedures: (i) subtracting each image from the first one taken immediately after the beginning of the humidification period; (ii) subtracting each image from the previous one. To facilitate the image interpretation, the same temperature scale was used in the color maps.

**Figure 10:**
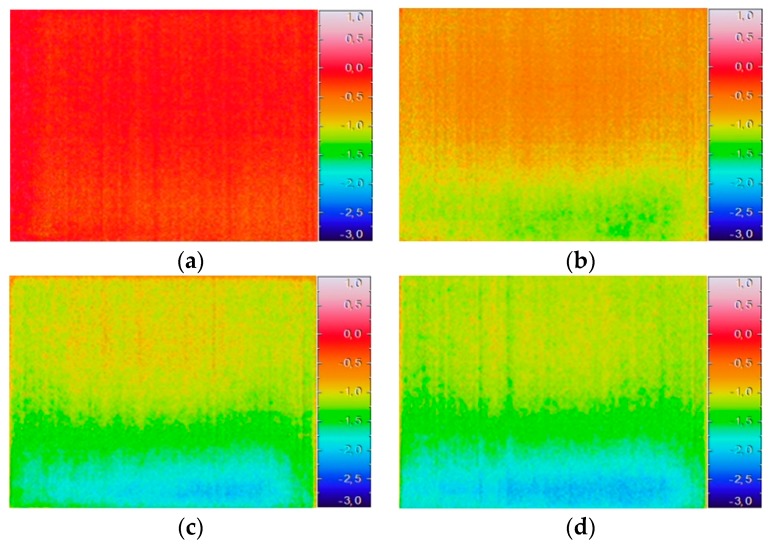
Image subtraction considering, as a reference, the first thermal image taken immediately after the humidification period began: (**a**) t = 0.5 h; (**b**) t = 2.5 h; (**c**) t = 9 h; (**d**) t = 24 h.

Figure 10 shows the results of the first procedure applied to four different moments: t = 0.5 h, t = 2.5 h, t = 9 h, and t = 24 h. In the first moment, after 30 min, although not obvious, the cooling effect was already observable in the bottom of the specimen (yellow pixels in Figure 10a). As the experiment proceeded, this effect was enhanced, especially at the bottom of the specimen. In fact, the temperature differences at the top of the specimen remained very similar, which indicates that moisture did not reach that level. At the bottom, temperature differences varied from approximately −1 °C in the first 30 min of humidification to −3 °C at the end of the measurement. However, the values tended to stabilize after the ninth hour, as no very relevant changes could be observed when comparing Figure 10c and Figure 10d.

This representation was very useful to highlight the evolution of moisture in the specimen, allowing the identification of the level reached by the raising water in each moment; it was possible to identify the areas where evaporation was more intense (blue regions in Figure 10).

**Figure 11:**
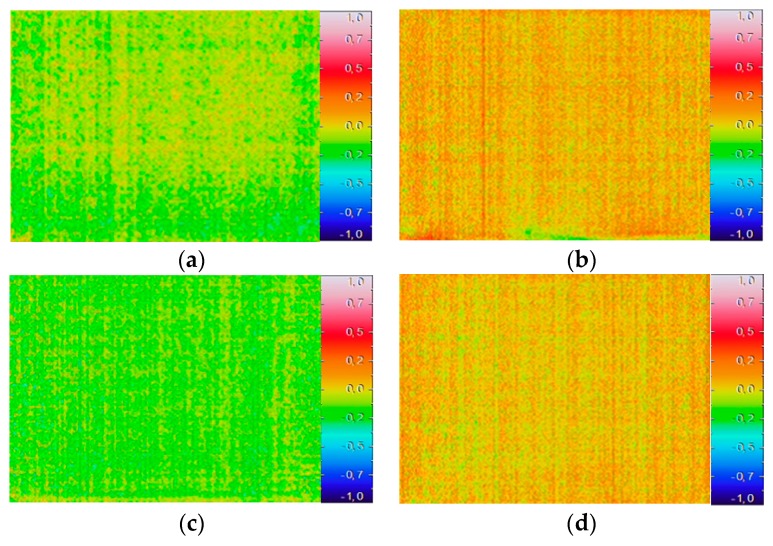
Image subtraction considering, as a reference, the previous image: (**a**) t = 0.5 h; (**b**) t = 2.5 h; (**c**) t = 9 h; (**d**) t = 24 h.

When assessing the phenomenon using, as a reference, the previous image (Figure 11), the result interpretation was not so obvious. This may be a surprising result because the statistical tests carried out in the sample reduction procedure, considering a significance level of 5%, indicated that the thermal images were significantly different. This suggests that the direct application of statistical tests can lead to inconsistent conclusions when evaluating the humidification phenomenon. Since the images were similar, the temperature variation was limited to a range of −0.5 °C to 0.2 °C, and no trend could be identified, whether over time or in the water level.

#### 3.2.3. Analysis Based on Index TI

**Figure 12:**
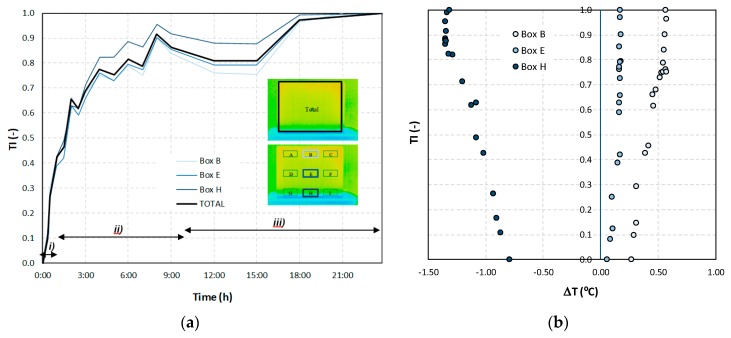
(**a**) Thermal index (*TI*) vs. time; (**b**) *TI* vs. ΔT.

The thermal index, *TI*, ranges between 0 and 1 and quantifies the cooling rate of the specimen. Figure 12a depicts the time-evolution of the index. In each thermal image, the index was calculated for the entire specimen and separately for each box. However, only the results of boxes B, E, and H are included in the figure to facilitate its interpretation, as these three boxes were representative of each level. The three phases already identified in the previous analysis were also visible in this representation. After the first hour, approximately 40% of the total cooling process already occurred. The overlapped lines of the graph confirmed that this temperature decrease was mostly due to the overall cooling of the specimen. Only after the first three hours was the effect of moisture on the surface temperature of the specimen clearly marked, as the value of index *TI* calculated for box H deviated from that obtained for the other two boxes, where the presence of water was lower and the cooling effect due to evaporation was less intense. 

Figure 12b shows the relationship between the thermal index, *TI*, and the temperature difference between each box and the entire specimen (Total). In boxes B and H, the temperature difference was almost constant during the experiment, indicating that the temperature in these boxes followed the trend of the entire specimen. On the other hand, the increasing difference found in box H until a TI of 0.8 was reached was further confirmation of the cooling effect at the bottom of the specimen. After reaching a TI of 0.8, the temperature difference tended to stabilize, suggesting that an equilibrium was achieved.

## 4. Conclusions

The main objective of this work was to deeply discuss the strengths and weaknesses of several methods to quantitatively assess the humidification phenomenon using IRT. For this purpose, a lightweight concrete specimen was partially humidified by the bottom surface for 24 h, and 193 thermal images were captured during that period. 

The methodology included a pre-processing phase for data reduction. A procedure was carried out to reduce the number of thermal images obtained during the experiment. The reduction was 88%, with a total of 23 thermal images being selected as relevant to characterize the time-variability of the temperature. The reduction procedure proved to be very important, as most of the images were, in fact, similar and, therefore, unnecessary to include in the numerical analysis of the phenomenon.

- The second phase of the methodology was the data processing. It was based on three methods: statistical descriptive analysis, image subtraction, and definition of a thermal index. The use of a box-plot representation over time considering the entire specimen was very interesting because the variability of the temperatures at certain moments of the experiment could be observed. It was found that the variability increased with the humidification process due to the increase of the wet area. The information provided by the outliers confirmed this conclusion, as their number tended to decrease throughout the test. Analyzing the box-plot representations of the boxes located in the central region of the specimen gave additional information regarding temperature variations at different levels, allowing an understanding of the water level achieved by the absorbed water at different moments of the experiment. In this case study, the temperature range in the lowest box, at different periods in time, pointed to the largest variations in the first hours, tending to stabilize at the end of the humidification.
- Image subtraction was a very interesting technique if the first image was used as a reference. When subtracting the previous image, the results were not so clear. This result confirmed that time is a key parameter to characterize the humidification phenomenon. Image subtraction is the most useful method if one intends to quantify the temperature differences due to moisture.
- The thermal index, *TI*, ranging between 0 and 1, allowed quantifying the cooling rate of the specimen. The three phases already identified through other methods were also highlighted by this index, as well as the confirmation of the cooling effect at the bottom of the specimen. However, more information could be obtained, as it was possible to identify that the effect of moisture on the surface temperature of the specimen only occurred after the first three hours.

## Figures and Tables

**Table 1:** Main characteristics of the lightweight concrete under study (measured).

Dimensions	0.28 × 0.21 × 0.075 m^3^
Dry density	1351 kg/m^3^ [34]
Water absorption coefficient	4.521 × 10^−3^ g/(mm^2^∙h^0.5^) [35]
Emissivity	0.91 [36]

**Table 2:** Main specifications of the infrared (IR) camera.

Measuring range	−20 °C to 100 °C
Resolution	0.06 °C at 30 °C, 60 Hz
Accuracy	±2 °C or ±2%
Detector	FPA (microbolometer)
Spectral range	8 and 14.0 μm
IFOV	1.2 mrad
Thermal resolution	320 × 240 pixels
Field of view	22° × 16°

FPA: Focal Plane Array; IFOV: Instantaneous Field of View.

**Table 3:** Statistical analysis of the temperature values (°C) of the thermal image taken at the end of the humidification period.

	**Box A**	**Box B**	**Box C**	**Box D**	**Box E**
Mean	16.9	16.9	16.9	16.5	16.5
Median	16.9	16.9	16.9	16.5	16.5
SD	0.06	0.06	0.06	0.09	0.09
Skewness coefficient	−0.16	0.07	−0.03	−0.19	−0.18
Maximum	17.0	17.1	17.1	16.8	16.7
Minimum	16.7	16.7	16.7	16.3	16.3
Box plot	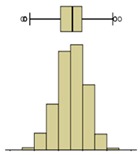	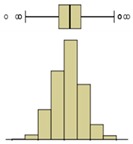	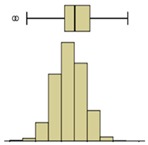	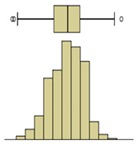	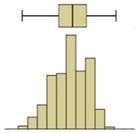
Histogram					
	**Box F**	**Box G**	**Box H**	**Box I**	**Total**
Mean	16.6	15.2	15.1	15	16.4
Median	16.6	15.2	15.1	15	16.6
SD	0.10	0.19	0.19	0.22	0.63
Skewness coefficient	−0.10	−0.01	−0.07	−0.05	−1.07
Maximum	16.9	15.6	15.5	15.5	17.2
Minimum	16.3	14.7	14.6	14.5	14.5
Box plot	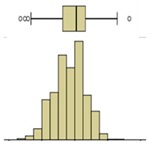	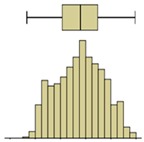	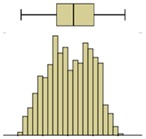	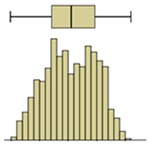	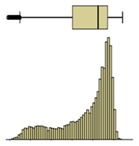
Histogram					
